# Use of bodily sensations as a risk assessment tool: exploring people with Multiple Sclerosis’ views on risks of negative interactions between herbal medicine and conventional drug therapies

**DOI:** 10.1186/1472-6882-14-59

**Published:** 2014-02-18

**Authors:** Lasse Skovgaard, Inge Kryger Pedersen, Marja Verhoef

**Affiliations:** 1 The Danish MS Society, Mosedalvej 15, 2500 Valby, Denmark; 2 Department of Public Health, University of Copenhagen, Øster Farimagsgade 5, 1014 Copenhagen, Denmark; 3 Department of Sociology, University of Copenhagen, Øster Farimagsgade 5, 1014 Copenhagen, Denmark; 4 NAFKAM, University of Tromsø, 9037 Tromsø, Norway

**Keywords:** Multiple sclerosis, Alternative treatment, CAM, Denmark, Contraindications, Negative interactions, Combination of conventional medicine and CAM, In-depth qualitative interviews, Mixed methods research

## Abstract

**Background:**

Most users of complementary and alternative medicine (CAM) combine it with conventional medicine. Recent risk assessment studies have shown risks of negative interactions between CAM and conventional medicine, particularly when combining herbal medicine and conventional drug therapies (CDT). Little is known about the way users consider such risks. The present paper aims to gain knowledge about this issue by exploring views on risks of negative interactions when combining herbal medicine and CDT among people with multiple sclerosis (MS).

**Methods:**

This paper draws on a qualitative follow-up study on a survey among members of the Danish MS Society. Semi-structured, in-depth qualitative interviews were conducted with a strategic selection from the survey respondents. The study was inspired by a phenomenological approach and emerging themes were extracted from the data through meaning condensation.

**Results:**

Four themes characterized the informants’ views on risks of negative interactions when combining herbal medicine and CDT: 1) ‘naturalness’ in herbal medicine; 2) ‘bodily sensations’ as guidelines; 3) trust in the CAM practitioner; 4) lack of dialogue with medical doctor.

**Conclusions:**

Generally, the combination of herbal medicine and CDT was considered by the informants to be safe. In particular, they emphasized the ‘non-chemical’ nature of herbal medicine and of their own bodily sensations as warrants of safety. A trustful relation to the CAM practitioner furthermore made some of them feel safe in their use of herbal medicine and CDT in combination. The informants’ use of bodily sensations as a non-discursive risk assessment may be a relevant element in understanding these issues.

## Background

Many people with Multiple Sclerosis (MS), as well as those with other chronic diseases, use complementary and alternative medicine (CAM^a^) treatments in the management of their disease [1]. Research has shown that prevalence of CAM use among people with MS ranges between 50-70% [2] and that CAM treatments are used for both specific and non-specific purposes [1,3-5]. Typically, people with MS who use CAM combine it with conventional treatments, although exclusive CAM users exist as well (the prevalence ranging from 10-30%) [6-9]. A recent Danish study has shown that 89.5% of the respondents among members of the Danish MS society, who had used CAM within the past year, had used it in combination with conventional treatments, most often conventional drug therapies (CDT) [10,11]. While it is known that different types of CAM treatments entail the risk of negative interactions when combined with CDT [12], it has been shown that CAM users in general do not reflect upon risks of adverse effects or negative interactions with conventional treatments [8,13,14]. CAM studies have in particular emphasized this tendency related to the use of herbal medicine [15,16].

Although the use of CAM and CDT in combination is known to be highly prevalent within the patient group, little is known about the way people with MS consider the risks of negative interactions from combining such treatments. Thus, this paper examines the question: *What characterizes the views on risks of negative interactions between herbal medicine and conventional drug therapies among members of the Danish MS Society, who combine these two types of medicine?*

## Methods

### The selection of informants

This paper is based on a larger sequential mixed methods study [17], using the results of a survey to inform and qualify the design of the interview study as presented below. Hence, the choice of research issue in this paper, as well as the selection of informants participating in the interview study, were based on the results of a preceding survey [10,11,18].

Results from this survey (n = 1865) among members of the Danish MS Society showed that 51.8% of the respondents had used CAM within the past twelve months, and that 89.5% of these responded that they had combined CAM with conventional treatments [10], mostly with CDT. For respondents combining CAM with CDT, dietary supplements and herbal medicine were particularly prevalent CAM modalities, pointing to interaction effects as an important safety issue for further investigation. We chose to focus on the use of herbal medicine due to the well known and well documented risk of negative interactions between herbal medicine and CDT [12].

We also used the results of the preceding survey to strategically select a group of informants. Statistical analyses of the survey data indicated that users of CAM and CDT in combination differed significantly from CAM non-users on five variables: they were more often <40 years, women, educated at bachelor level or higher, belonging to a household with high income and affected by multiple diagnoses [19]. We used three of these five variables as inclusion criteria: age, gender and level of education, leaving out income and prevalence of multiple diagnoses. The choice of leaving out the income variable was based on the fact that income was indicated as average income per person in household and thereby saying less about the individual informant. Leaving out the prevalence of multiple diagnoses was based on our wish to focus primarily on the informants’ use of treatments linked to MS. Hence, as a result of this strategic selection, our group of informants was limited to young women with a high level of education, who had reported combined use of CDT and herbal medicine within the past twelve months, and who had accepted in the survey to be contacted for an interview. This selection process provided us with a group of 13 informants. Semi-structured interviews were conducted with 11 informants, as two informants declined to participate in the study. The informants were guaranteed anonymity and pseudonyms have been used for their names. Their characteristics are presented in Table 1.

**Table 1:** Characteristics of informants (all women)

**Name**	**Age**	**Years of education**	**Treatments used within the past year**
Ann	38	16	Prescription medicine, non-prescription medicine, herbal medicine, supplements of vitamins and minerals, supplements of oils, physical therapy, massage, psychology/psycho therapy, acupuncture.
Bertha	39	18	Non-prescription medicine, herbal medicine, supplements of oils, yoga, physical therapy.
Cecilia	38	16	Non-prescription medicine, herbal medicine, supplements of vitamins and minerals, special diet, Tai Chi, physical therapy.
Doris	36	17	Prescription medicine, non-prescription medicine, herbal medicine, supplements of vitamins and minerals, supplements of oils, homeopathy, physical therapy, massage, therapeutic horse back riding, chiropractics, healing, kinesiology, hypnosis, meditation.
Elinor	39	20	Prescription medicine, non-prescription medicine, herbal medicine, supplements of vitamins and minerals, supplements of oils, massage, psychology/psycho therapy.
Fay	37	18	Non-prescription medicine, herbal medicine, supplements of vitamins and minerals, supplements of oils, special diet, yoga, chiropractics.
Gina	38	17	Prescription medicine, non-prescription medicine, herbal medicine, supplements of vitamins and minerals, supplements of oils, special diet, physical therapy.
Heather	39	15	Prescription medicine, non-prescription medicine, herbal medicine, supplements of vitamins and minerals, special diet, homeopathy.
Ingrid	33	17	Non-prescription medicine, herbal medicine, acupuncture.
Jane	33	17	Prescription medicine, non-prescription medicine, herbal medicine, supplements of vitamins and minerals, supplements of oils, special diet, yoga, Qi Gong, massage, reflexology, meditation.
Kylie	31	16	Prescription medicine, non-prescription medicine, herbal medicine, supplements of vitamins and minerals, physical therapy, acupuncture.

In Denmark, only biomedical research projects can be approved by a Committee of Health Research Ethics. This study was registered at the Danish Data Protection Agency. Written consent for participation was obtained from all participants in the study.

### The study design

The study has been inspired by a phenomenological approach in its focus on exploring how a specific group of patients make sense of their experiences and the meaning they give to experiences within a certain context. Thus, the overall interest has been to gain an insider perspective of the way different treatments are chosen, used or foregone by the informants [20,21]. Fade argues that the phenomenological approach is relevant within health research when the aim is to explore perceptions of a given situation or phenomenon within a specific group of patients or practitioners [22]. Smith et al. stress that the phenomenological approach is highly suitable when attempting to gain an insider perspective of a given phenomenon being studied [23], in this case the patients’ views on risks regarding the use of herbal medicine and CDT in combination. In this study, the phenomenological approach has been applied as suggested by Hycner [24], whose steps for phenomenological analysis have been used as a guideline, combined with the method of meaning condensation as described by Kvale [25] for the identification of themes.

The interviews were semi-structured, allowing for narrative aspects of the informants’ reflections to have a strong presence. However, the main focus of the interviews was on the informants’ use of CDT and herbal medicine in combination, and the data presented in this article are mainly based on the informants’ views on the specific issue of risk. The issue of possible disadvantages by combining CDT with herbal medicine was introduced at the end of the interview by the interviewer, if it had not been broached automatically – directly or indirectly - during the interview.

The interviews initially addressed the informants’ experiences with their life with MS in general, asking questions such as: “Would you tell me about your life with MS?” or “In your experience, what affects the development of your MS?”. The interviews also specifically addressed the issue of CAM and herbal medicine, asking questions such as: “Why do you use herbal medicine?” and “what were your experiences using herbal medicine (or other CAM modalities)?”. Hence, the informants were initially asked to relate broadly how they had experienced the impact of various factors/interventions/treatments on their health. In connection to different factors/interventions/treatments they were then asked to relate which elements they assumed had been relevant with regards to the outcomes experienced, being positive or negative. This way of gathering knowledge about informants’ general treatment assumptions was inspired by previous use of program theory – a tool to facilitate the articulation of participants’ basic assumptions of how a given intervention leads to a given outcome [26-29].

The length of the interviews varied from 35–65 minutes. The interviews were audio-recorded and subsequently written up as in-depth summaries with illustrative quotations. Themes were extracted from the data material through meaning condensation [25]. Meaning condensation entails an abridgement of the meanings expressed by the informants into themes. Each interview summary was firstly read through in order to get a sense of the whole. Thereupon, meaning units as expressed by the informants were determined and thematized into overall themes. Finally, themes were identified that related to the entire interview study [25]. Illustrative quotations were extracted from the audio-recordings to illustrate the informants’ in-vivo articulation of themes.

## Results

From the analyses, four themes emerged in connection to the issue of possible risks of negative interactions between the two types of medicine: 1) the element of ‘naturalness’ in herbal medicine; 2) the use of ‘bodily sensations’ as guidelines; 3) trust in the CAM practitioner and 4) lack of dialogue with medical doctor. In the following, these four themes will be presented, each accompanied by selected, illustrative quotations.

### The element of naturalness in herbal medicine

Most of the informants referred to the aspect of ‘naturalness’ in herbal medicine, indicating a clear distinction between the ‘chemical’ aspect of CDT and the ‘non-chemical’ nature of herbal medicine.

*“I became convinced that natural medicine was the right way to go… Probably mainly because there have been so many stories in the media about the prescribed, conventional medicine – how it can be harmful in all kinds of ways. (…) I have this blind faith, that this is not the case with herbal medicine, because it’s a naturally occurring thing.”* (Bertha, age 39)

*“I read a book and thought: Eating those plant-caps can’t hurt. And I don’t think it has.”* (Fay, age 37)

The two informants cited above expressed a confidence that herbal medicine would not harm them due to its naturalness. One informant referred to CDT as ‘artificial’, indicating a certain genuineness of herbal medicine based on its occurrence in nature.

*“I prefer taking medicine that exists in nature. I feel more certain that it will not harm my body. When it’s not artificial.”* (Jane, age 33)

The chemical aspect of CDT was by several informants linked to a risk of harming the body. Toxicological aspects linked to herbal medicine were only addressed by one informant in a passing remark:

*“Natural remedies somehow seem healthier, because they exist in nature. I realize that there can be certain issues. Plants can be poisonous of course, but still.”* (Heather, age 39)

This informant expressed some concern regarding possible risks entailed by use of herbal medicine, but at the same time she insisted on the superiority of natural remedies compared to CDT with regards to health. Another informant mentioned a possible risk linked to an excessive intake of herbs and a third informant addressed the issue of possible negative interactions between CDT and herbal medicine. The remaining eight informants expressed a perception of herbal medicine as safe to use, emphasizing the element of naturalness as an important aspect.

Most of the informants did not give the impression of having previously reflected extensively upon risks of negative interactions between herbal medicine and CDT. One informant responded, when asked by the end of the interview about the issue of risks of combining CDT with herbal medicine:

*“The herbal medicine I take, I can’t imagine that causing any problems. But maybe it does, I won’t rule that out (…) I think, that herbal medicine is very natural… so no, I haven’t worried about side effects or negative effects or the like. Maybe I will be proven wrong, but no, I haven’t.”* (Elinor, age 39)

Another informant said:

*“Yeah, well, I suppose they could* [interact]. *But I sort of feel that it’s nature. Uhm, so it’s not something I am afraid of. And I haven’t noticed in my own body that any of the herbal medicine I’ve taken has made me ill. I haven’t experienced that.”* (Heather, age 39)

When being asked about the issue, these informants referred to the naturalness of herbal medicine or to the absence of experienced negative effects as warrants of safety. A third informant referred to the herbs as a natural part of a healthier living, linking the use of herbal medicine to the use of healthy food (e.g. vegetables) in general:

*“I don’t really have any experiences with that* [negative effects of herbal medicine]*. I’ve really barely considered it. Living healthier can’t harm… healthier food I mean. But also vegetables and herbs and the like.”* (Gina, age 38)

One informant expressed a specific concern regarding possible negative interactions and underlined the importance of dialogue with the medical doctor:

*“I only do it* [use herbal medicine] *after consulting the doctor, precisely because, well you can’t say…. There could be interactions between some herbal medicine and that sort of thing, and the medicine I take. So I prefer to check first, whether it’s okay to do, right?”* (Ann, age 38)

This informant was the only informant to address directly the issue of possible negative interactions between herbal medicine and CDT before the issue of possible disadvantages was brought up by the interviewer at the end of the interview.

### The use of bodily sensations as guidelines

Closely linked to the aspect of naturalness and artificiality of herbal medicine and CDT, several informants mentioned the importance of being aware of bodily reactions to the medicine used. One of the informants expressed it in this way, emphasizing the applicability to both alternative and complementary medicine:

*“Well, I feel that, if it doesn’t work, you give it up. It’s a matter of testing it and assessing how it feels (…) You have to pay attention to how your body feels, regardless of what you’re taking. And regardless of whether it’s conventional or alternative.”* (Fay, age 37)

The necessity of personal assessment was underlined by several informants, referring to the value of the individual experience. Two informants said:

*“I think that there might well be* [negative] *side-effects – particularly from the excipients. Certainly you can take too many vitamins and herbs, and then you have to take a break. It’s about paying attention to how it feels.”* (Jane, age 33)

*“Well, if I suddenly noticed some side-effects that I hadn’t noticed before, because I, say, started using acupuncture, or taking some specific supplements or herbs, I would think about whether it was right for me. Like I said, it’s one thing that acupuncture is good for me, but it might not be good for everyone. Health is extremely specific to the individual.”* (Ingrid, age 33)

In the latter quotation, the informant emphasizes the aspect of individual characteristics as an additional reason for paying attention to the personal, bodily sensations. Although indicating an element of surprise, when asked to relate to the issue of possible risks, several informants referred to use of bodily sensations as an important warrant of safety:

*“Yeah, well, I suppose they could* [interact] (…) *I haven’t noticed in my own body that any of the herbal medicine I’ve taken has made me ill. I haven’t experienced that.”* (Heather, age 39)

*“I haven’t talked to anybody about there being a problem with mixing. I really haven’t considered that. Maybe I should look into it further. I’m actually realizing that I often feel a bit hung-over after eating raw garlic.”* (Elinor, age 39)

As illustrated by these quotations, the risk of negative interactions entailed by the combination of herbal medicine and CDT was not necessarily an aspect that the informants previously had reflected upon. However, they emphasized the use of bodily sensations as a kind of non-discursive reflection – an ongoing, immanent assessment of treatments used.

### Trust in the CAM practitioner

In addition to the aspect of bodily sensations as a warrant of safety, several of the informants expressed a certainty that their CAM practitioner would be aware of possible negative interactions. Two informants said:

*“And he* [the practitioner] *knows full well (…) he sees many, many sclerosis patients at his clinic, so he has a lot of experience with it, and he would never give me something he thinks might make me sick, I just don’t think he would. So I fully trust him, and it’s not the case that I take all kinds of different strange things. I don’t just buy some herbal medicine and eat it.”* (Doris, age 36)

*“But it’s all through her* [the practitioner] *– I would never take natural medicine I didn’t know a lot about. So she takes care of it, I don’t decide what I take, and I barely know what it is… it’s some Chinese stuff. I take what she tells me to take (…) I haven’t had any side effects from it…. so no, I don’t think so. I haven’t* [considered specific possible disadvantages linked to an intake of herbal medicine].*”* (Jane, age 33)

These quotations illustrate that some of the informants also used their CAM practitioner in their decision making and referred to his/her professional experience as a warrant of safety. An experience several of the informants did not trust their medical doctor to have.

### Lack of dialogue with medical doctor

Several informants indicated the absence of interest or knowledge from their medical doctor as one reason for not engaging in a dialogue with him/her about possible disadvantages when combining herbal medicine and CDT. Some informants expressed that this absence had left them without incentive to engage in such dialogue:

*“No, because my doctor is dreadfully old-fashioned. So I definitely do not want to discuss it with him. I don’t, unfortunately. It will not get me anywhere.”* (Bertha, age 39)

*“Well, I don’t really feel that it’s something where I have to bring my doctor into my decision about doing some things and not doing others (…) I search the web a lot, but a lot of times it’s difficult to navigate. And I think, that if you were to talk to the doctor about it, it would be impossible.”* (Elinor, age 39)

Several informants emphasized that their attempts to engage in dialogue had not been fruitful due to insufficient seriousness from the medical doctor:

*“I’ve told them about the different things I take, so they know about it, but they don’t really care that much. “I see, I see”, is all they say. My former physician thought it was very fascinating and very interesting, but then, unfortunately, he left. And these new ones, they’re not as…. “I see, I see”, they say. I don’t think they believe in it at all.”* (Doris, age 36)

*“I’ve told them that I use different types of alternative medicine and herbal medicine, and I can just go ahead and do that, they say… so it hasn’t been a dialogue.”* (Gina, age 38)

*“I’ve been told that I have to be cautious when it comes to mixing herbs, and supplements and conventional medicine. But I’ve never been told why, or exactly under which circumstances.”* (Ingrid, age 33)

As illustrated above, one of the informants had received some information from her medical doctor about possible risks of negative interactions, but not specific information that she found useful. Several of the informants expressed a reluctance to engage in communication with their medical doctor about their use of herbal medicine due to a belief that such communication would not be successful.

As illustrated by the four themes that characterize the informants’ views on risks of negative interactions when combining herbal medicine and CDT, such combination was in general considered by the informants to be safe. They entrusted the safety to the naturalness of the herbal medicine, to their personal ongoing bodily sensations as well as to the expertise of their CAM practitioners. The majority of the informants did not feel encouraged to engage in communication with their medical doctor about the issue.

## Discussion

The analyses of the interview data showed that the informants had not previously reflected extensively on possible risk of negative interactions when combining herbal medicine and CDT. Previous studies among CAM users in general have indicated that the perception of CAM as risk-free is prevalent [14-16,30,31] and that CAM users often consider CAM to be low-risk due to its ‘natural’ basis [13,14,30]. A few studies have investigated users’ beliefs and/or perceptions regarding the safety of use of herbal medicine specifically, indicating a similar trend [31-34]. The findings of the present study support these findings, indicating that the informants regard the combination of herbal medicine and CDT as safe, not least based on the element of naturalness in herbal medicine. The results of the present study add to the existing knowledge by emphasizing bodily sensations as an important warrant of safety from a user’s point of view. In the following, a few aspects of this issue will be discussed, suggesting as a perspective that the informants’ way of addressing the issue of safety in a non-discursive way through the use of bodily sensations may contribute to understanding communicative challenges between patient and medical doctor regarding CAM.

### The informed body

Researchers have emphasized that the use of CAM may represent a health strategy in which patients try to regain or gain control over their disease [35-37]. Studies of people with chronic disease show that building embodied knowledge and developing the body as a personal capacity may be a way to gain control and may play a substantial role in patients’ feeling of being able to navigate their way through a chronic course of disease [38-40]. Furthermore, studies of CAM treatments have shown that CAM practitioners work with patients’ active awareness, including bodily awareness [26,38], and that use of CAM may reside in the development of a learning potential that strengthens patients’ capacity to intervene in relation to their own disease [35,38]. Thus, when the informants in our study refer to bodily sensations as a warrant of safety, this way of assessing risk may be linked to the development and/or optimization of a bodily learning potential. A study among members of the Danish MS society, investigating treatment assumptions among exclusive users of CAM, indicates that the users regard the development of personal, bodily experiences as an important part of personal health enhancement [41].

The aspect of bodily sensation may be relevant to the discussion of self-screening and self-diagnosis among the lay public as an important aspect of a modern health-orientated consumer culture as e.g. presented by Chrysanthou [42]. Chrysanthou emphasizes the importance of “the informed body” as a tool of navigation for the individual health consumer. In this perspective, the importance of bodily sensations emphasized by the informants, rather than reflections on possible negative side-effects or interactions with CDTs may indicate that other types of information than traditional evidence-based information, i.e. more individual, experience-based, bodily embedded types of information - are seen as valuable.

### Lack of communication with medical doctor

In many of the studies addressing the issue of safety regarding use of herbal medicine, lack of communication with a medical doctor appears a major concern. Studies have indicated that users of herbal medicine often do not inform their primary care provider about their use of CAM [32-34]. An Irish study from 2008 indicated that this issue is also relevant within MS treatment as only 25% of patients using CAM at an Irish neurological clinic had informed their medical doctor about use of CAM [43]. Several studies emphasize the importance of improving doctors’ communication and openness regarding CAM treatments – especially regarding the safety aspect related to use of herbal medicine [15,34,44]. This aspect of non-disclosure is in several cases explained by the medical doctor’s expected lack of interest/knowledge of herbal medicine or patients’ fear of negative response from medical doctor [32,44]. The informants in the current study emphasize those two aspects as well, supporting the results of previous studies. In addition, it may be relevant to include the aspect of bodily sensation as a risk assessment tool in the discussion on communicative challenges between patient and medical doctor regarding use of CAM as it may constitute an issue of discordance in fundamental perceptions regarding the usefulness of patient experiences. Hence, differences in views on the epistemological value of the patients’ bodily sensations as a warrant of safety may complicate communication between patient and doctor regarding treatment risks linked to use of CAM as well as the proper way of assessing such risks.

### Strengths and limitations of the study

The mixed methods frame has offered an emergent research approach where the qualitative study was designed based on what was learned from the initial quantitative phase. Although based on the statistical analyses of survey data, the process of strategic selection of informants has also entailed the risk of selection bias when choosing the inclusion criteria for informant selection. Thus, it cannot be ruled out that other themes would have emerged if the informants had been selected randomly among all users of herbal medicine and CDT in combination.

The sample size of 13 informants, of which 11 accepted to participate in the study, was a result of the strategic selection. A group of 11 informants is not necessarily sufficient to secure full data saturation and it can not be ruled out that additional themes could have emerged from a larger sample. However, the aim of this study was not to perform analyses based on fully saturated data material, but to explore possible themes within a strategically selected group of informants. Since the four explored themes presented in the Results section were consistent among most of the informants, we believe that a certain saturation has been achieved, although further studies should be recommended.

The use of an overall phenomenological approach in the study was useful in supporting the achievement of an insider perspective of the informants’ experiences and their views related to their use of different treatments. The phenomenological focus on the way meaning is attached by the informants to their various choices was especially relevant in analyzing the absence of explicit reflections among the informants on the risk of combining herbal medicine and CDT, as it provided insight into other aspects of CAM use, for instance the use of individual bodily sensations, to which the informants ascribed profound value.

The act of partly structuring an interview entails the risk of certain issues being elicited by the interviewer rather than arising spontaneously from the informant. One cannot rule out that the element of structure in the performance of the interviews in this study may have limited the narrative aspect of the interviews and thereby also contributed to a certain curtailment of the types of experiences articulated by the informants. At the same time, the use of a certain structuring has allowed for the informants’ views upon the specific issue of risk to be ensured in the interviews. Furthermore, the aspect of structuring has shown to be of relevance within the specific patient group, as the risk of cognitive challenges is prevalent among people with MS [45], entailing a risk of lack of structure in free dialogue.

## Conclusion

The informants in this study generally considered the combination of herbal medicine and CDT to be safe. The issue of potentially negative interactions between CDT and various CAM treatments, not least herbal medicine, has been addressed by several studies within the past decade. These studies indicate as well that patients who wish to include CAM treatments as a supplement to their conventional drug therapy do not necessarily consider epidemiologically estimated risks entailed by such combination as being relevant. In this study, the informants emphasized the ‘naturalness’ in – and ‘non-chemical’ nature of – herbal medicine and their individual bodily sensations as important warrants of safety. They also emphasized a trustful relation to their CAM practitioner. However, we need more knowledge about patients’ use of bodily sensation as a non-discursive risk assessment. We also need more knowledge about the ways in which differences in views on the value of such assessments might affect patient-doctor communication. The issue of bodily sensations as presented in an increasing number of studies may point to the fact that individual, experience-based types of information are seen as valuable by CAM users and that the development of embodied knowledge may be of growing relevance for people in coping with a chronic illness.

## Endnote

^a^In this study, we followed the definition of CAM suggested by the American National Center for Complementary and Alternative Medicine (NCCAM), who define CAM as a group of diverse medical and health care systems, practices, and products that are not generally considered part of conventional medicine. Dietary supplements and herbal medicine are in this definition a part of CAM.

## Competing interests

The authors declare that they have no competing interests.

## Authors’ contributions

LS drafted this manuscript. IKP and MV helped conceive, design and provided critical edits to this manuscript. All authors read and approved the final manuscript.

## Pre-publication history

The pre-publication history for this paper can be accessed here:

http://www.biomedcentral.com/1472-6882/14/59/prepub
